# Microscale Heterogeneity Explains Experimental Variability and Non-Linearity in Soil Organic Matter Mineralisation

**DOI:** 10.1371/journal.pone.0123774

**Published:** 2015-05-19

**Authors:** Ruth E. Falconer, Guillaume Battaia, Sonja Schmidt, Philippe Baveye, Claire Chenu, Wilfred Otten

**Affiliations:** 1 SIMBIOS School of Science, Engineering and Technology, Abertay University, Dundee, United Kingdom; 2 Bioemco, AgroParisTech, Université Paris-Saclay, Thiverval-Grignon, France; 3 Laboratory of Soil and Water Engineering, Department of Civil and Environmental Engineering, Rensselaer Polytechnic Institute, Troy, New York, United States of America; Chinese Academy of Sciences, CHINA

## Abstract

Soil respiration represents the second largest CO_2_ flux from terrestrial ecosystems to the atmosphere, and a small rise could significantly contribute to further increase in atmospheric CO_2_. Unfortunately, the extent of this effect cannot be quantified reliably, and the outcomes of experiments designed to study soil respiration remain notoriously unpredictable. In this context, the mathematical simulations described in this article suggest that assumptions of linearity and presumed irrelevance of micro-scale heterogeneity, commonly made in quantitative models of microbial growth in subsurface environments and used in carbon stock models, do not appear warranted. Results indicate that microbial growth is non-linear and, at given average nutrient concentrations, strongly dependent on the microscale distribution of both nutrients and microbes. These observations have far-reaching consequences, in terms of both experiments and theory. They indicate that traditional, macroscopic soil measurements are inadequate to predict microbial responses, in particular to rising temperature conditions, and that an explicit account is required of microscale heterogeneity. Furthermore, models should evolve beyond traditional, but overly simplistic, assumptions of linearity of microbial responses to bulk nutrient concentrations. The development of a new generation of models along these lines, and in particular incorporating upscaled information about microscale processes, will undoubtedly be challenging, but appears to be key to understanding the extent to which soil carbon mineralization could further accelerate climate change.

## Introduction

Soil respiration has been acknowledged as the second largest CO_2_ flux from ecosystems to the atmosphere, and researchers have recognized that a small change in soil respiration could contribute significantly to a further rise in atmospheric CO_2_, increasing the threat of a ‘runaway climate’ scenario [1]. In spite of that, understanding the environmental controls of soil respiration, and especially of whether soil respiration is likely to be enhanced by rising soil temperatures and changes in the frequency of rainfalls, remains contradictory.

Convergent evidence suggests that this limited understanding, and the ensuing reality that the outcomes of experiments in this area remain largely unpredictable, stem from the fact that we are missing something essential, which occurs in soils at the spatial scale at which microorganisms operate, and that existing models are based on assumptions, which need to be alleviated. Experiments typically involve samples at the core-scale (5 cm height and diameter), characterized in terms of bulk properties, without taking into account heterogeneities that are known to exist at micro-scale with regard to the distribution of microorganisms, nutrients, or the geometry of the pore space in which both microorganisms and nutrients co-evolve [2–7]. Models of the fate of soil organic matter likewise ignore details of the heterogeneity of microbial populations and their physiology, overwhelmingly describing microbial processes by 1^st^ order kinetics relations, which often conflict with experimental results that exhibit high variability [1], a clear influence of microbial physiology [2], and a marked non-linear character [3]. For example, experiments with fungi have shown their mode of spread and growth switches from invasive to non-invasive, which can be interpreted as autocatalytic and conservative behaviours. However, further work has shown in spatially explicit soil, plant and nutrient environments that this behaviour is affected not just by fungal physiology but also by environmental cues including the density, quantity and heterogeneity of spatially distributed resources [4] or the pore network of a soil environment through which fungi spread [5]. We argue that accurate predictions can be obtained only when considering the physiology of microorganisms and their interplay with carbon distribution within a physically heterogeneous environment. Fortunately, tremendous technological advances, in terms of imaging, simulation [8–11] and experimental techniques, now allow unprecedented quantification of the microscale heterogeneity of the physical, chemical and biological characteristics of soils. This information can be used to develop a new generation of models to better describe the fate of soil organic matter, which in turn help predict the macroscopic outcomes associated with various scenarios of microscopic heterogeneity.

In this general context, a first objective of this article is to develop a spatially-explicit model of the fate of soil Particulate Organic Matter (POM), a fraction of the soil organic matter that typically has residence times ranging from 1 to 20 years in temperate areas [6]. The model relies on information about the 3-dimensional pore space of soils obtained via X-ray computed tomography. It accounts for physical and chemical processes at the micro-scale, as well as for the growth and metabolism of fungi, whose significance in POM decomposition is well acknowledged, especially in forest ecosystems [13–15]. Using this model, a second objective described in the following is to assess the extent to which the amount and spatial distribution of soil POM influences the fungal colony growth and spread, and the amount of CO_2_ that evolves over time.

## Materials and Methods

The soil samples on which this study was based were obtained from land belonging to French National Institute for Agricultural Research (INRA) and permission was granted. Integration of soil physical, chemical and biological components requires three components outlined in turn below: (1) characterisation of the pore volume; (2) location of POM at the pore/solid interface; (3) formulations of SOM-microbial feedback model.

### Soil sampling and X-ray CT scanning

Soil was sampled from the surface 15 cm of a Eutric Cambisol (FAO; 17% clay, 56% silt and 27% sand) in the “La Cage” long-term trial at the INRA research centre of Versailles (France). Soil was sieved to aggregates <5 mm and packed at a bulk density of 1.4 g cm^-3^. The soil samples were scanned with a Nikon Metrology X-Tek HMX CT scanner at energies of 100 kV 131 mA, with 3010 projections. A Varian Paxscan 2520 V detector, a 225 kV X-ray source (Nikon Metrology X-Tek Systems Ltd, Tring, UK) and a focal spot of 5 mm were used. A molybdenum target was used with a 0.25 mm thick aluminium filter to reduce beam hardening. NIKON software CT Pro v2.0 was used for reconstruction using a filtered back-projection algorithm. The voxel resolution was 50 microns. 2D cross sections (top view) from the 3D volumetric images of soil samples were saved as image sequences (slice thickness = voxel size) using VGStudio MAX v2.1 software. These images were cropped to a size of 200 × 200 × 200 voxels. The cropped images were converted to binary images using Indicator Kriging to separate the volume into a pore and a solid phase, and to produce the 3D structures within which POM was placed and simulations were conducted [7].

### Placement of POM in the 3D soil structures

Since the true distribution of POM is unattainable using benchtop XRay μCT, a prescribed amount of Particulate Organic Matter (POM), defined as being larger than 50 μm, was distributed along the pore-solid interface, assuming homogenous or heterogeneous coverage controlled by magnitude and degree of aggregation (clustering).

A method was developed to produce random spatial distributions of POM within the 3D soil structure, adhering to global constraints, such as the POM (g) inputs per cm^3^ of soil, and the particle size class distribution of POM. This permits detailed testing of microbial response to contrasting POM distributions that can be described by general characteristics obtained from soil analysis, such as quantity of POM and size fraction of POM. POM is distributed randomly (from a uniform random distribution) in the solid matrix at the pore-solid interface, according to the following set of simple rules and recognizing that POM is part of the solid phase. POM is generated from a central voxel randomly chosen in the set of solid voxels that have at least one porous neighbour. The POM aggregate is defined as the set of solid voxels located not further than a given distance from this centre. In the case of a flat surface POM aggregates would have a semi-spherical shape. Each voxel in the 3D volume can therefore have one of three states: (i) pore volume, (ii) inert solid phase, or (iii) POM. The number of particles of POM (N_aggreg_) contained in the sample is set equal to the ratio between the volume of POM and the volume of a sphere that has the specified diameter d_pom_:

$${\text{N}}_{\text{aggreg}}=\left({\text{m}}_{\text{pom}}/\rho_{\text{pom}}\right)/\left(\pi .{\text{d}}_{\text{pom}}{}^{3}/6\right),$$ Eq. 1

where m_pom_ is the specified amount of POM, and *ρ*_pom_ is the mass density of POM. In the same manner, the number voxels of POM voxels (N_pom_voxel_) in the sample required is given by:

$${\text{N}}_{\text{pom}\_\text{voxel}}=\left({\text{m}}_{\text{pom}}/\rho_{\text{pom}}\right)/\left({\text{h}}^{3}\right),$$ Eq. 2

where h is the voxel edge length (50μm).

### Scenario Modelling

Having developed a flexible way to place POM within a given soil architecture, one can then investigate the interplay between soil carbon dynamics and colony dynamics. The scenarios will investigate the effect of POM inputs and size class and distribution on fungal colonisation and activity. The amounts of POM and certain POM size class distributions are obtained from experimental data [8]. The POM amounts were varied between 0–5% sample weights (g/g) [9]. The allocation of POM into size classes has been described in numerous papers [10,11]. POM is defined as being greater than 50 microns in size and is typically grouped into 51–200 microns and 201–2000 micron classes. The same size classes were adopted in this study with the inclusion of a fine POM size being equal to 50 microns [10,11]. Following [12], Minkowski functionals were used to characterise the distribution of POM in space, including measures for POM volume fraction and % accessible POM (Table 1). POM volume fraction calculates the fraction of POM voxels with respect to total number of voxels in the sample. % accessible POM calculates the fraction of POM voxels that are located on a solid-pore interface—hence are accessible—, with respect to the total number of POM voxels.

**Table 1:** Description of the scenarios developed within the paper.

	POM input		POM distributions and characteristics				
scenario	% POM (w/w)	POM (mg/g)	mg POM with diameter 50 μm	mg POM with diameter 51–200 μm	mg POM with diameter (>200 μm)	POM volume fraction	% Assessable POM (stdev)
1	0	0	0	0	0	0	0(0)
2	0.1%	1.4	1.4	0	0	0.0007	2.02 (0.05)
3	1%	14	14	0	0	0.0067	19.89 (0.15)
4	2%	28	28	0	0	0.0134	39.95 (0.28)
5	3%	42	42	0	0	0.02	60.12 (0.23)
6	4%	56	56	0	0	0.0268	80.06 (0.18)
7	5%	70	70	0	0	0.0335	100 (0)
8	3%	42	0	0	42	0.0200	49.95 (6.03)
9	3%	42	35.5	4.53	1.07	0.0196	56.73 (0.28)
10	3%	42	1.07	4.53	35.5	0.019	51.06 (1.75)

POM size classes (= 50, 51–200 & >200) were informed by literature and by POM found in the field [8]. Scenario 9 reflects the distribution of POM sizes observed in [8]. The measure of POM volume is provided together with the % accessible POM on pore/solid interface with average and standard deviation for each scenario. The % of accessible POM is crucial as it determines the accessibility to organic matter. Replicates are 5 except for scenarios 8–10 where n = 15.

### Modelling Scenario 1—Assessing the effect of POM inputs

In the first scenario, we tested the impact of randomly distributed POM within the range of 0–5% C (g/g soil). We assume all POM to be of the smallest size (= 50 μm), therefore all POM is contained within a single voxel layer between the solid and pore phase (as the voxel size of the data was 50 μm). The conditions are given in scenario 1–7 in Table 1. Each simulation was repeated 5 times with different realisations in POM distributions, resulting from the random placement of POM on the pore/solid interface (except for when POM = 0 and 5% when there is no and complete coverage of solid/pore interface with POM).

### Modelling Scenario 2—Assessing the effect of POM size distribution

To determine the effect of spatial distribution and POM size on evolved CO_2_, the quantity of POM was held constant while the POM sizes and their distributions varied. A POM content of 3% was selected as this was where a switch appeared to occur from non-invasive (limited spatial extent) to invasive growth (filled the accessible pore volume available). To represent POM size classes of 50, 51–200 microns and 201–2000 microns, we selected average diameters for each class of 50, 100 and 1000 microns, respectively to be used in our method for POM distribution as explained below.

Four scenarios were compared: (1) a scenario where all POM is of a small size (1 voxel in diameter, 50 μm) (identical to scenario 5 in Table 1), (ii) a scenario with all POM of a large size (scenario 8 where POM diameter is 1 mm (20 voxels) (Table 1)), (iii) and (iv) assume distributions of POM with scenario iii skewed towards the smaller size and with a distribution consistent with experimentally obtained POM size class distributions, and scenario (iv) with a similar distribution but now skewed to the larger POM sizes (Table 1).

In both scenarios the spatial distribution of POM is unknown, and therefore was randomly distributed within the solid phase as described in section Placement of POM.

### Microbial SOM-Microbial Feedback Model

**Fig 1:**
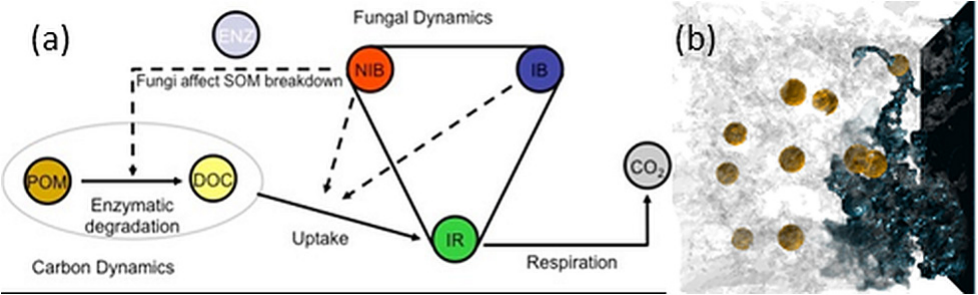
Biophysical modelling of soil organic matter dynamics. a) The components of the physiological based fungal model describing mineralisation of organic matter in soil. State variables are circles and arrows indicate transformations. Dotted lines represent processes driven by Michaelis Menten (MM) kinetics. b) A snap shot of fungal biomass (blue) initiated from the right hand plane and distributed through the pore volume (transparent gray pixels) in relation to POM (brown pixels) at t = 150hrs; the fungal biomass shown is the sum of the three types (NIB, IB, IR) as in 1a.

The key physiological processes of the fungal growth model are uptake, biomass recycling (inter-conversions amongst three fungal biomass types required for biomass recycling), and respiration associated with uptake of Dissolved Organic Carbon (DOC) (Fig 1A). Internal resource is translocated through the colony and is converted into biomass—hyphal tips (NIB) or an insulated biomass (IB), the latter possessing a reduced uptake capacity of DOC. NIB is converted into IB over time which is analogous to rigidification of hyphal structure [13]. NIB and IR spread via diffusion, being constrained by pore space and biomass respectively. Extensions to the model included the formulation of SOM-fungal feedback embedded in a soil context. The SOM-microbial feedback can be described as how the type, size and distribution of POM affect the partitioning of biomass between hyphal tips (NIB) or biomass for conservation (IB), which affects enzyme production and therefore the subsequent rate of carbon decomposition. The feedback is mediated via enzyme production which is assumed to be in proportion to the density of hyphal tips (NIB). The enzymes transform SOM (POM → DOC), and the DOC can subsequently be utilised by hyphal tips (NIB) and converted into internal resources (IR). Owing to the large density of carbon in POM compared to fungal biomass, POM degradation is assumed to be driven by a high affinity process. The degradation rate is therefore proportional to the enzyme concentration. Enzymes are released from the hyphal tips (NIB) and are assumed to have a limited range of diffusion. POM degradation follows a first order kinetic process with respect to amount of NIB. As the simulation proceeds (simulation run equivalent to 3 weeks real time), POM amounts remain nearly constant, which is consistent with persistence time of POM in soils e.g. months for particulate organic matter such as maize [6]. Once DOC is produced it can diffuse freely in the porous media but it is also available for fungal uptake. Uptake is described by a Monod equation. The metabolic cost for NIB is represented by a loss of IR proportional to NIB amount that results in the production of CO_2_. Again the sustainability cost for IB is considered to be negligible.

The system of equations can be written as:

∂POM∂t=−NIB.Vpom.POMKPOM+POM

∂DOC∂t=∇.(Ddoc.∇DOC)+NIB.(VpomPOMKPOM+POM−ϕb.Vdoc.DOC(Kdoc+DOC))

∂IR∂t=∇.(Dir.∇IR)+NIB.(δu.ϕb.Vdoc.DOC(Kdoc+DOC)−ξ)+NIB.(βn.π−αn.πθ)+IB.(βi.π−αi.πθ)

∂NIB∂t=∇.(Dnib.∇NIB)−NIB.(βn.π−αn.πθ)−ζ.NIB

∂IB∂t=-IB.(βi.π−αi.πθ)+ζ.NIB

∂CO2∂t=NIB.(ξ+(1−δu).ϕb.Vdoc.DOC(Kdoc+DOC))

Where α_n_, α_i_, β_n_, β_i_ and θ are the recycling parameters, *π* stands for the internal resource concentration in the mycelium (*π* = IR/(IB+NIB)) and *ζ* is the insulation parameter (see [13]). D_doc_, D_ir_ and D_nib_ are the diffusion parameters respectively for DOC, IR and NIB which characterise the spread of the fungus through the pore volume as it finds new C resources. V_pom_, K_pom,_ V_doc_ and K_doc_ are the Michaelis-Menton (MM) degradation and uptake parameters. *ξ* and *δ*_*u*_ are metabolic costs. They are commonly referred to as maintenance and growth respiration, respectively. *ϕ*_*b*_ is a function of ∑ B = NIB + IB + IR bounded between 0 and 1. It limits the total amount of fungal biomass in each single voxel to a maximal value B_max_ being such as *ϕ*_*b*_ = 0 when ∑ B = B_max_ and *ϕ*_*b*_ = 1 when ∑ B = 0. This function can be given by the empirical expression *ϕ*_*b*_ = 1 - ∑ B/B_max_.

### Model parameterisation and calibration

The model was parameterised for *Rhizoctinia solani* Kühn, a soil pathogen and saprotrophic organism, and parameters were estimated via a literature review and sensitivity analysis, and were defined in terms of carbon transfer and flow [12]. Previous research has established that the fungal growth model, based on established theory and microbial parameters, is robust. Briefly, some parameters, governing specific physiological processes, were estimated via an extensive literature search and experimentation. Parameter values that could not be determined in this manner were identified, and the consequences of uncertainty in parameter values were assessed through sensitivity analysis. This analysis showed that predictions of biomass yield and extent are most sensitive to uncertainties in the recycling processes.

In the microbial-SOM model presented in this study, the microbial component now interacts with POM. The parameters associated with the breakdown of POM into DOC are unfortunately unknown. Values associated with POM inputs and size distributions can be obtained from the literature [8–9] or measured experimentally. The main parameter uncertainty is in the distribution of POM, as this cannot reliably be classified using existing segmentation methods applied to soils. The introduced parameters associated with decay of POM into a more accessible form (DOC) are presented in Table 2 together with their values. As an initial starting point we assumed the Michaelis-Menten (MM) parameters (V_pom_ and K_pom_) to be an order of magnitude smaller than V_Doc_ and K_Doc,_ and the latter values were determined and provided in [14].

**Table 2:** Additional parameter values for Microbial-SOM model.

Parameter	Description	Values
V_POM_	MM POM decay parameter	0.1*V_DOC_
K_POM_	MM POM decay parameter	0.1*V_DOC_
POM_AMOUNT (m_pom_)	POM input	0–5% (g/g)
POM_DIAM (d_pom_)	POM diameter	50, 100 and 1000 μm
POM SIZE DISTRUBTIONS	% of POM attributed to each size class	See Table 3
POM spatial distribution	Location of POM in soil sample	Randomly distributed at pore-solid interface

The main parameters of the microbial model are obtained from experimental data and literature research as described in [14].

**Table 3:** Scenarios i—iv describing the POM size distributions explored.

	0.05 (mm)	0.1 (mm)	1.0 (mm)
Scenario i	84.5%	10.8%	4.7%
Scenario ii	4.7%	10.8%	84.5%
Scenario iii	84.5%	10.8%	4.7%
Scenario iv	4.7%	10.8%	84.5%

The selected Michaelis-Menten (MM) parameter values (V_POM_ and V_DOC_) produced reasonable simulated outputs in terms of matching slow degradation of POM and cumulative respired CO_2_ amounts, consistent with the literature [8–9]. The values for POM_QUANTITY, POM_DIAMETER, and POM size distributions were derived from the literature and experimentation as justified in “Scenario Modelling” section.

The spatial location of POM within the soil is unknown therefore it is distributed randomly adhering to the criteria stipulated in section “Placement of POM”. Uncertainty in how POM is distributed can be assessed via system outputs (CO_2_ respired) propagated from uncertain inputs. We will use the variability in model output (cumulative CO_2_ respired) as a criterion to identify uncertainty and importance associated with the POM distributions.

## Results

### Integration of soil physical, chemical and biological components

**Fig 2:**
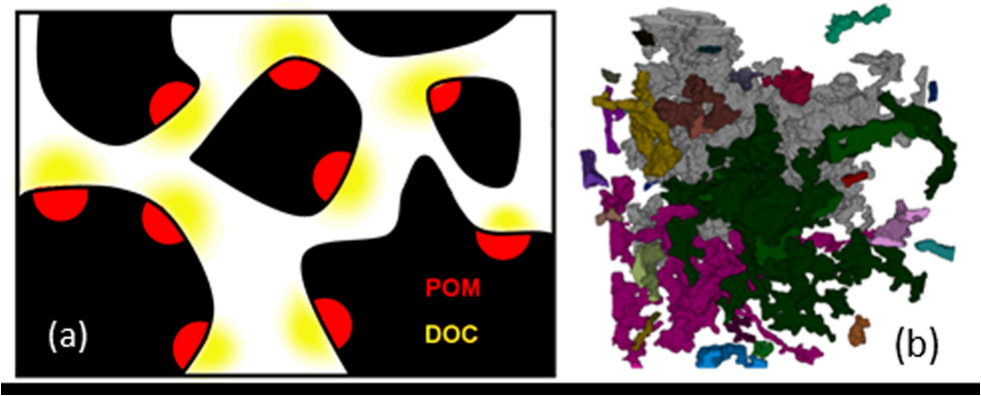
Soil heterogeneity affecting fungal invasion and CO_2_ production. a) A 2D schematic of the soil illustrating the distribution of POM (brown pixels) solid (black pixels) and pore (white pixels) phases. Enzymes produced by fungi transform POM into DOC (yellow) which is subsequently taken up by fungi and used for growth and CO_**2**_ production b) The complex pore geometry of the soil sample used in this study is visualised using X-ray μCT. The sample shown has a pore volume of 10% (pores > 50μm) and a pore-solid surface area of 74.7 mm^2^. Different colors represent disconnected pore volumes, and the volume is dominated by two large disconnected connected clusters (gray and green).

On the basis of the X-ray μCT data, the pore geometry visualised at a resolution of 50 μm is depicted in Fig 2. The visible porosity, which includes pores larger than 50 μm, was 10%, with a pore-solid surface area of 74.7 mm^2^. The pore volume was dominated by two large interconnected pore clusters and many smaller disconnected clusters, represented by different colors in Fig 2B. The large disconnected clusters imply that much of the pore volume can potentially become fully occupied by fungi if sufficient carbon is available for growth and spread.

### CO_2_ production relates in a non-linear way to C content in soil

**Fig 3:**
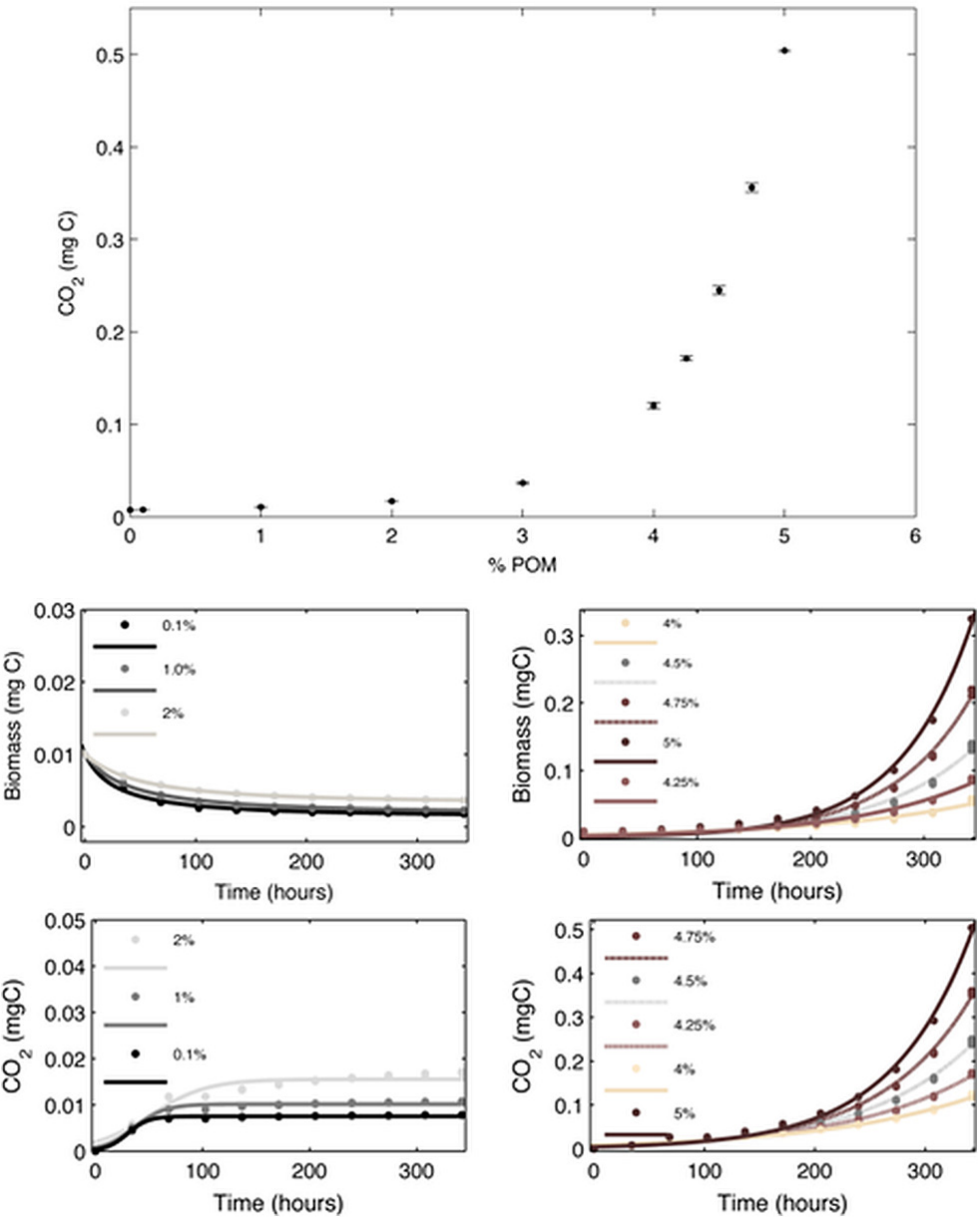
Fungal dynamics affected by increasing POM input on the soild/pore interface. a) non-linear response in the CO_**2**_ evolution to POM input at end of the simulation (340 hours) with N = 5 (standard deviation is represented by error bars). (b,c) Simulated biomass and the cumulative amount of CO_**2**_ produced (d,e) during the first 15 days after inoculation of the soil samples. Two distinct dynamics are observed with fungal biomass either increasing or decreasing with time, depending on the POM input. This difference in fungal growth also results in distinct differences in the amount of CO_**2**_ produced, which either levels off (with the rate of CO_**2**_ production approaching 0) (d) or continues to increase (e).

Simulations assessed the impact of POM, distributed at the pore-solid interface, on fungal growth and evolved CO_2_. With increasing mass of POM there is an increase in the number of POM voxels on the pore/solid interface, ranging from zero to all interface voxels being occupied with POM (Table 1) affecting POM accessibility for fungal growth. Simulations show a non-linear relationship between POM input and evolved CO_2_ (respiration) with only a small degree of variability amongst replicates (Fig 3A). Depending on the POM input, two distinct responses of fungal behaviours were observed with fungi either decreasing in biomass (conservative growth for low POM inputs, Fig 3B) or increasing (autocatalytic growth for high POM inputs, Fig 3C). Above a POM content of 3% g/g, the cumulative evolved CO_2_ follows an exponential trend with time (resulting from autocatalytic growth of fungi). Below a POM content of 3% a levelling off of cumulative CO_2_ is observed (Fig 3D and 3E).

### Micro-scale distribution of POM causes substantial variability in respiration

**Fig 4:**
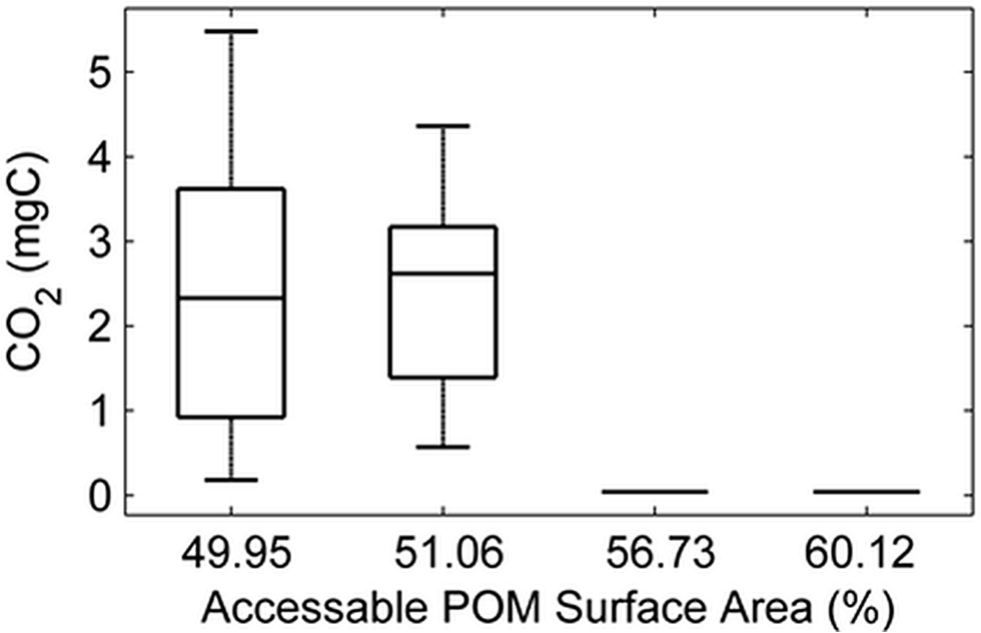
Boxplots show simulated variability in CO_2_ production for samples with identical POM content of 3%. Y axis is evolved CO_**2**_ per cm^3^ of soil. X axis represents the scenarios reflecting different POM distributions i) is a scenario where all POM is of a large size, (ii) a scenario where POM distribution is skewed towards large POM diameters (iii) a scenario where distribution is skewed towards fine POM (small diameters) and (iv) assume distributions of fine POM (50 micron diameter) rows 1–4 of Table 4). From left to right the % of accessible POM on the pore-solid interface is 49.95, 51.06, (N = 15) 56.73 & 60.12 (N = 5) %. Samples differ in the way POM is distributed within the soil sample assuming different size classes (Table 1).

The threshold value of POM that promoted a transition from conservative to autocatalytic growth was further investigated. This threshold was where 60% pore/solid interface voxels were POM, which corresponds to a POM content of 3% (g/g). The investigation involved the spatial allocation and clustering of POM by manipulating the POM size classes (Table 1). In particular the POM size classes were varied ranging from fine POM (1 voxel or 50 μm) to aggregated POM (20 voxels or 1000 μm). The realised POM distribution was characterised by geometric measures such as volume and % accessible surface area (see Table 1 in MM). The micro-scale heterogeneity of POM had a large impact on mean CO_2_ production as well as the variability. For samples with an identical POM content of 3%, the cumulative mean CO_2_ produced ranged from 0.034 to 2.38 mg C per cm^3^. This is approaching a striking 100 fold difference in CO_2_ production from samples with identical bulk POM content and porosity resulting from differences in the spatial distribution of POM at the solid-pore interface at the microscale. In addition, and in particular for samples where POM has relatively large particles (>200 μm) substantial variability is observed between replicated samples. Due to large variability in simulation results a total of 15 replicates were run. Interestingly, and perhaps counter intuitive, higher respiration is observed for a population containing larger POM size classes despite that these will have a smaller cumulative surface area (Fig 4) accessible to fungal colonisation.

**Table 4:** The conditions selected for model simulations with POM particle size distribution altered to determine effect on microbial growth and activity.

% POM	% Assess. POM	Total sample POM (mg)	mg POM with diameter 50 μm	mg POM with diameter 51–200 μm	mg POM with diameter 1 mm	Mean CO_2_ (stdev)	Mean Biomass (stdev)
3%	49.95 (n = 15)	42	0	0	42	2.32 (1.66)	1.40 (1.07)
3%	51.06 (n = 15)	42	1.07	4.53	35.5	2.38 (1.07)	1.34 (0.66)
3%	56.73 (n = 5)	42	35.5	4.53	1.07	0.03 (0.00)	0.01 (0.00)
3%	60.12 (n = 5)	42	42	0	0	0.04 (0.00)	0.01 (0.00)

## Discussion

Central to the development of an understanding of SOM decomposition by fungi is an understanding of fungal ecology, influenced by the physical environment, type and distribution of substrates. Many carbon decomposition models, which do not have an explicit consideration of microbial ecology, do not observe a nonlinear response of respiration to carbon inputs, most likely due to the assumptions of the underlying models i.e. decomposition is affected by carbon pool only (1st order) [15] as opposed to the interplay amongst microbial physiology, the structured environment in which these organisms reside and C distributions. These results therefore show that the assumptions made in soil C models critically affect the predictions of CO_2_ in relation to available C and that when underlying heterogeneity is considered a different relationship can emerge. Our second main finding which shows that substantial variability in POM turnover rate occurs at identical bulk properties is far reaching; it demonstrates that under certain conditions the bulk measurement (e.g. average porosity and average C content) of soils is insufficient to predict the production of CO_2_. This variability is attributed to microscale heterogeneity and microbial dynamics. Although this is only one component of the C cycle in soils it suggests that a different approach to larger scale soil C models based upon trends derived from models with explicit consideration of microbial dynamics and heterogeneity may be required to improve the current predictions of the roles of soils in climate change models. Finally, it highlights the importance of obtaining experimental data at appropriate spatial scales.

Two modes of collective fungal behaviour, conservative and autocatalytic, both sensitive to POM inputs, are observed. This behaviour is determined by a combination of the spatial distribution of POM within the pore volume as this conditions the accessibility to organic matter, and of the intrinsic physiological processes that regulate fungal growth, spread and biomass partitioning within the colony. This affects the efficiency of POM decomposition within the pore volume. When conservation occurs, the little POM that is available is used for maintenance, leaving a lesser amount available for enzyme and biomass production. Biomass becomes insulated over time and spread is insufficient to access new POM; this further limits enzyme production, uptake of DOC and hence production of CO_2_. The stationary phase is realised fast in comparison with autocatalytic behaviour. For the autocatalytic behaviour the system is yet to be limited by carbon and fungi are in an exponential growth phase. This critical switch in collective behaviour of fungi is consistent with other experimental and theoretical work examining the effect of spatial distribution of C sources in a 2D environment where a small change in density [16] or connectivity of pore network [17] made fungi switch from non-invasive to invasive spread. Non-invasive spread is when the spatial extent of fungi is limited, whereas invasive spread occurs when the colony spreads and proliferates in the available pore space.

The large differences observed in POM turnover rate for identical bulk C inputs are a consequence of including the microbial physiology. Interestingly the larger POM pieces are metabolically more efficient to decompose than many separate smaller carbon hot spots voxels, an, at first surprising result. The separated smaller POM pieces do not invoke such a rapid decomposition of POM as enzyme production and DOC uptake is lower and the decomposition rate is hampered further by colony scale insulation (colony conservation). This colony-scale insulation acts to convert active (NIB) biomass into non-active form (IB) therefore reducing the colony’s ability to produce enzymes. By altering the parameters that govern the physiological processes of colony growth, reflecting different, ecological strategies other results, i.e. more biomass and activity with smaller chunks of POM, may be obtained. This would likely be achieved by faster diffusion rates and less metabolic costs thus promoting more explorative colony behaviour as opposed to the observed exploitative behaviour. Here the fungal parameters used were calibrated for *Rhizoctania solani* Kühn based on literature values and parameter estimation [14].

## Conclusions

Predictive modelling of soil carbon dynamics is a key factor in future climate change prediction. It has been suggested that uncertainties in predictions can be associated with a lack of representation of microbial dynamics at appropriate spatial scales in current soil carbon models. Our results show how biophysical interactions predict non-linear responses to carbon inputs in soil and explain the huge variability observed for samples with similar bulk characteristics. The results highlight the need for the development of a novel class of soil carbon models and a significant departure from existing SOM decomposition models to form a comprehensive understanding of soil-carbon responses to changing abiotic factors, as this response depends on efficiency of C usage by soil microbes. Further efforts should now focus on the effect of other ecological strategies of fungi and on considering microbial diversity, chemical composition and physical characteristics to identify the key drivers of C dynamics, and to upscale the observed patterns of behaviour to determine core-scale model parameters (CO_2_ efflux, biomass yields) from measures of structure and carbon distribution. These patterns of behaviours can then be used in larger scale C dynamics models. Major advancements in soil science will be made if it is possible to translate micro-scale soil processes to macro-scale soil properties.
